# Sodium variability is associated with increased mortality in severe burn injury

**DOI:** 10.1186/s41038-017-0098-4

**Published:** 2017-11-06

**Authors:** Soman Sen, Nam Tran, Brian Chan, Tina L. Palmieri, David G. Greenhalgh, Kiho Cho

**Affiliations:** Department of Surgery, Division of Burn Surgery, University of California Davis, 2425 Stockton Blvd. Suite 718, Sacramento, CA 95817 USA

**Keywords:** Sodium, Burns, Critical illness, Ions, Hypernatremia, Hyponatremia

## Abstract

**Background:**

Dysnatremias are associated with increased mortality in critically ill patients. Hypernatremia in burn patients is also associated with poor survival. Based on these findings, we hypothesized that high plasma sodium variability is a marker for increased mortality in severely burn-injured patients.

**Methods:**

We performed a retrospective review of adult burn patients with a burn injury of 15% total body surface area (TBSA) or greater from 2010 to 2014. All patients included in the study had at least three serum sodium levels checked during admission. We used multivariate logistic regression analysis to determine if hypernatremia, hyponatremia, or sodium variability independently increased the odds ratio (OR) for death.

**Results:**

Two hundred twelve patients met entry criteria. Mean age and %TBSA for the study was 45 ± 18 years and 32 ± 19%. Twenty-nine patients died for a mortality rate of 14%. Serum sodium was measured 10,310 times overall. The median number of serum sodium measurements per patient was 22. Non-survivors were older (59 ± 19 vs. 42 ± 16 years) and suffered from a more severe burn injury (50 ± 25% vs. 29 ± 16%TBSA). While mean sodium was significantly higher for non-survivors (138 ± 3 milliequivalents/liter (meq/l)) than for survivors (135 ± 2 meq/l), mean sodium levels remained within the laboratory reference range (135 to 145 meq/l) for both groups. Non-survivors had a significantly higher median number of hypernatremic (> 145 meq/l) measurements (2 vs. 0). Coefficient of variation (CV) was significantly higher in non-survivors (2.85 ± 1.1) than survivors (2.0 ± 0.7). Adjusting for TBSA, age, ventilator days, and intensive care unit (ICU) stay, a higher CV of sodium measurements was associated with mortality (OR 5.8 (95% confidence interval (CI) 1.5 to 22)). Additionally, large variation in sodium ranges in the first 10 days of admission may be associated with increased mortality (OR 1.35 (95% CI 1.06 to1.7)).

**Conclusions:**

Increased variability in plasma sodium may be associated with death in severely burned patients.

## Background

Sodium metabolism is usually tightly regulated at the cellular and organ level. Alterations in plasma sodium may be a consequence of iatrogenic interventions such as intravenous fluid administration and medication or may portend altered physiology and may be a marker for a rapid decline in health. For critically ill patients, dysnatremias are among the most common electrolyte abnormalities. Dysnatremias are also associated with increased mortality in critically ill patients [1]. Sodium is an extracellular cation, and it is the primary and most osmotically active cation in the body [2]. As a result, both cellular and plasma concentrations are tightly regulated. This regulation is mediated through the kidney via several neurohormonal mechanisms. Disruption of this regulation results in abnormal sodium levels that reflect abnormalities in serum water content and circulating plasma volume [3]. These changes can have profound effects on a patient’s physiology resulting in increased morbidity and mortality.

For critically ill patients, the abnormal sodium concentrations arise from several sources. First, administration of sodium containing fluids contributes to the development of dysnatremias. Most of these fluids are given intravenously and account for the majority of daily sodium load [4]. Additionally, while sodium administration for critically ill patients are generally above the daily recommended level, the osmotic concentration of the sodium containing fluids also contribute to altered plasma sodium concentration [5]. The use of hypotonic fluids can increase total body water causing the development of hyponatremia. In contrast, hypertonic fluids can result in a relative loss of total body water resulting in hypernatremia [6]. Second, a number of medications can contribute to the development of dysnatremias. A number of commonly used medications such as thiazide diuretics, proton-pump inhibitors, and antipsychotics impair renal salt metabolizing function and cause salt wasting producing hyponatremia. Other medications such as loop diuretics, antifungals, and osmotic cathartic agents (lactulose) can cause loss of water and volume depletion resulting in hypernatremia [7]. Third, alterations in renal function contribute to the development of dysnatremias. These alterations may be in response to the changing physiology as a result of injury, inflammation, or infection resulting in an increased absorption of water by the kidneys [8]. The alterations may also reflect disrupted neurohormal regulation resulting in the inability of the nephrons to absorb water and concentrate the urine [9]. Declining renal function as a result of either pre-existing renal impairment or acute renal injury can also result in altered renal sodium and water filtration [10, 11]. Fourth, medical conditions such as diabetes, congestive heart failure, cirrhosis, hypothyroidism, and hypopituitarism can all underlie altered plasma sodium concentration. Additionally, severe cutaneous injuries such as burn injuries and blast injuries result in the loss of both water and sodium [12].

For burn patients, hypernatremia that occurs within a few days of injury may be associated with increased risk of death [13]. Additionally, early hypernatremia may effect wound healing and skin grafting success [14]. Most of the studies investigating the impact of dysnatremias on survival in critically ill patients focus on admission or early development of either hyper or hyponatremia [5, 15]. However, more recent evidence suggest that more subtle variations of plasma sodium concentration may have an impact of outcomes for critically ill patients [16]. Moreover, sodium variability during a patient’s hospital course may be a marker of physiologic decline [17, 18]. Incorporating alterations in sodium levels with other clinical indicators of physiologic status may improve the accuracy and precision of determining a severely burned patient’s clinical course. Based on these recent findings, we hypothesized that sodium variability will be independently associated with an increased risk of death in severely burned patients. Our aim for this study was to determine if sodium variability, hypernatremia, and hyponatremia are associated with increased mortality in severely burned adult patients.

## Methods

We performed a retrospective cohort study involving adult (18 years and older) patients who suffered a 15% total body surface area (TBSA) burn injury from 2010 to 2014. We obtained permission to perform the study from the University of California Davis Institutional Review Board. All data were obtained from the patient’s electronic health record. Data collected included the following: age, gender, TBSA of burn injury, discharge disposition, mortality, intensive care unit (ICU) length of stay, and ventilator days. We also collected all plasma sodium measurements available during the entire hospitalization period for all patients included in the study. We included patients who met the above criteria and who has at least three plasma sodium measurements during their hospitalization. We excluded patients with a known previous history of chronic renal failure or dialysis dependent renal failure.

All patients included in the study were resuscitated under a standard burn resuscitation protocol. This included calculation of fluid requirements for acute burn resuscitation using the Parkland formula. All patients were resuscitated using lactated Ringer’s solution or normal saline. Hypertonic saline was not used for resuscitation. All patients received standard glucose monitoring. Glucose levels were maintained per protocol between 80 and 150 mg/dl using either intermittent insulin administration or an insulin infusion. All measurements of plasma sodium were performed in the hospital central laboratory. Reference range for normal sodium measurements was 135 to 145 meq/l. For the plasma sodium measurements, we determined the mean sodium for each patient and the mean standard deviation (SD) of sodium for each patient. Hypernatremic measurements were defined as any sodium measurement greater than 145 meq/l. Hyponatremia was defined as any sodium measurement less than 135 meq/l.

Data was analyzed using R statistical package (www.r-project.org). For all analyses, significance levels were set at a *p* < 0.05. Normality of the data was tested using the Shapiro-Wilk test of normality. Comparisons of means between two groups of normally distributed data were performed using the student’s *t* test. For non-normally distributed data, the Mann-Whitney *U* test was used to compare differences between groups. The chi-square test was used to assess association between discrete categorical variables. Variability of sodium measurements was assessed by the coefficient of variation (CV) which is the SD divided by the mean multiplied by 100. Multivariate logistic regression analysis was performed to determine associations between categorical outcome variables and independent continuous and categorical predictor variables. For our multivariate model, the outcome variable was mortality. The input variables were determined in a stepwise subtraction method. Final model determination was made with the following criteria: input variables that were independently and significantly associated with the outcome variable and the Akaike Information Criterion (AIC). There were issues of collinearity in the regression model and the best-fit model did not include hypernatremia or hyponatremia. The occurrence of hypernatremia was rare even in non-survivors, and CV can take into account the potential association of sodium dysnatremia in a larger population of burn patients. We used only significant variables into the model. We then removed variables from the model that did not reach statistical significance with respect to predicting mortality. For variables that had “zero” values, we included them in the models using the same criteria for *p* value. We used the AIC to determine if the variables should continue to remain in the model. We confirmed that the remaining variables in the model were the best fit through determining the AIC. The AIC for the model presented was 40, which was lower than that for the model with the starting set of variables. This left us with the following input variables for the model: age, %TBSA, ventilator days, ICU days, and mean SD of plasma sodium. None of these variables contained any zero or null values.

## Results

### Patient demographics

Out of the 212 patients entered into the study, 29 patients died (14%). The mean age and burn severity were approximately 45 years and 32%TBSA, respectively. Median ventilator days and length of ICU stay were 2 and 22 days. There were four patients admitted with pre-injury chronic renal failure, 38 patients who developed acute renal failure during hospitalization, and two who required dialysis during hospitalization. Plasma sodium was measured 10,310 times for a median of 22 plasma sodium measurements per patient (Table 1). Overall, 41% of plasma sodium measurements were below 135 meq/l and only 1.6% of measurements were above 145 meq/l.

**Table 1:** Patient demographics of overall study

Patient demographic	
Males	164
Females	48
Lived	183
Died	29
Burn area (%TBSA)	32 ± 19
Age (years)	44.6 ± 18
ICU stay (median days)	22 (15–39)
Mechanical ventilation (median days)	2 (0–15)
Acute renal failure	38
Chronic renal failure	4
Dialysis	2
Sodium measurements (median)	22 (12–51)
Mean sodium concentration (meq/l)	135.5 ± 2.4
Mean sodium standard deviation	2.9 ± 1.1

All mean values are mean ± standard deviation, all median values are median (interquartile range)

*TBSA* total body surface area, *ICU* intensive care unit, *meq/l* milliequivalents per liter

### Non-survivors suffer from more hypernatremia and higher sodium variability

As expected, non-survivors were older (59 vs. 42 years, *p* < 0.0003) and suffered on average a larger burn injury (50% vs. 29%TBSA, *p* < 0.0001). Non-survivors also had a significantly longer median length of mechanical ventilation (17 vs. 1 day, *p* < 0.00001). There was no difference in median length of ICU stay (18 vs. 20 days, *p* = 0.23). Non-survivors suffered from significantly higher rates of acute renal failure than survivors (72% vs. 9.2%, *p* < 0.0001). Non-survivors had a significantly higher median number of plasma sodium measurements (35 vs. 20, *p* = 0.03) and also had a significantly higher mean sodium concentration (138 meq/l vs. 135 meq/l, *p* < 0.01) compared to survivors. The median number of hyponatremic sodium measurements was not different between non-survivors and survivors (13 vs. 9, *p* = 0.79). In contrast, the median number of hypernatremic plasma sodium measurements was significantly higher in non-survivors (2 vs. 0, *p* < 0.01). Additionally, the median percentage of hypernatremic plasma sodium measurements was also significantly higher in non-survivors (6% vs. 0%, *p* < 0.01). Hyponatremia was more frequent in survivors (40% vs. 20%, *p* < 0.01). The SD of the measured sodium values was calculated for each patient as an indication of sodium variability. Comparisons of the CV of the measured sodium values showed that non-survivors had significantly higher sodium variability (CV = 2.85 ± 1.1) compared to survivors (CV = 2.0 ± 0.7, *p* < 0.01) (Table 2).

**Table 2:** Survivors vs. non-survivors

Patient demographic	Survivors	Non-survivors	*p* value
Males	142	22	
Females	41	7	1
Burn area (%TBSA)	29 ± 16	50 ± 25	< 0.01
Age	42 ± 16	59 ± 19	< 0.01
ICU stay (median days)	20 (13–38.5)	18 (8–36)	0.23
Mechanical ventilation (median days)	1 (0–10)	17 (7–36)	< 0.01
Acute renal failure	17 (9.2%)	21 (72%)	< 0.01
Sodium measurements (median)	20 (12–44.5)	35 (23–89)	0.04
Hyponatremic measurements (median)	9 (4–17.5)	13 (2 to 21)	0.79
Hypernatremic measurements (median)	0 (0)	2 (0–7)	< 0.01
Frequency of hyponatremia (median)	40% (24–62%)	20% (11–40%)	< 0.01
Frequency of hypernatremia (median)	0% (0%)	6% (0–8.6%)	< 0.01
Mean sodium concentration (meq/l)	135 ± 1.9	138 ± 3.4	< 0.01
Mean sodium standard deviation	2.7 ± 1	4 ± 1.5	< 0.01
Coefficient of variation	2.0 ± 0.7	2.85 ± 1.1	< 0.01

Patient demographic. All mean values are mean ± standard deviation, all median values are median (interquartile range)

*TBSA* total body surface area, *ICU* intensive care unit, *meq/l* milliequivalents per liter

### Large ranges of plasma sodium measurements in the first 2 weeks after burn injury are associated with mortality

We examined the range of sodium measurements following admission to determine if there was a time-dependent association between changes in plasma sodium and death. We analyzed sodium measurements over 5-day intervals from admission to death or discharge and calculated a range. The mean range of sodium in the first 5 days was 7.8 ± 3 meq/l for survivors and 10 ± 5 meq/l for non-survivors (*p* < 0.009). From days 6 to 10, the mean range for survivors was 4 ± 2.5 meq/l and for non-survivors was 6 ± 2.7 meq/l (*p* < 0.000001). After day 10, there were no significant differences in ranges between survivors and non-survivors. We then performed a multivariate regression model that adjusted for age and TBSA and found the increased sodium range 6 to 10 days after admission was associated with an increased risk of death (OR 1.35 (1.06–1.7).

### Plasma sodium variability is independently associated with mortality

A multivariate model was constructed to determine independent predictors for mortality in this patient sample. Increased age (OR 1.17, *p* = 0.002), larger burn size (OR 1.2, *p* = 0.0006), and increased length of mechanical ventilation (OR 1.3, *p* = 0.009) are all independent significant risk factors for death. Increased length of ICU time (OR 0.72, *p* = 0.0006) increases the likelihood of survival. This probably reflects the fact that survivors had an overall longer hospital course than non-survivors. For the effect of plasma sodium on mortality, increased CV, reflecting plasma sodium variability, is an independent risk factor for death (OR 5.8, *p* = 0.01) (Table 3).

**Table 3:** Multivariate logistic regression model for risk factors of death

Variable	Odds ratio	95% CI	*p* value
TBSA	1.2	1.1–1.3	0.0006
Age	1.17	1.1–1.3	0.002
Mechanical ventilation days	1.3	1.1–1.5	0.0009
Length of ICU stay	0.72	0.6–0.9	0.0006
Coefficient of variation	5.8	1.5–22	0.01

Multivariate logistic regression model assessing for independent risk factors that are associated with death

*TBSA* total body surface area, *ICU* intensive care unit, *CI* confidence interval

## Discussion

Dysnatremia is a significant risk factor for mortality in critically ill patients [19]. A recent study investigating the effects of dysnatremia on mortality in patients with community-acquired pneumonia found that both hyponatremia and hypernatremia were significantly associated with death. Furthermore, the investigators found that extreme alterations of sodium concentrations have a higher hazard ratio for mortality [20]. The effect of hypernatremia on mortality may be more impactful than hyponatremia. Several retrospective studies have shown a mortality rate range of 30% to 48% for patients with severe hypernatremia (> 150 meq/l) [21]. In critically ill burn patients, hypernatremia is a common condition and can occur in up to 11% of severely burned patients. The most common etiology underlying the development of hypernatremia is loss of total body water through insensible losses and sepsis [22, 23]. A recently published study by Stewart et al. shows that similar to critically ill medical patient, hypernatremia is a risk factor for mortality in burn patients. This study investigated an effect of dysnatremias on mortality in all burn patients (mean TBSA of 9%) [24]. In contrast, our investigation examined the association of dysnatremia on mortality in patients with larger burn injuries (mean TBSA of 32%). The Stewart study showed that hyponatremia occurred in 6.8% of patients and hypernatremia occurred in 9.9% of patients. Our study showed a higher rate of dysnatremia with over 42% of the sodium measurements either above or below the normal range. Our study also showed that hypernatremia is more common in patients who died and that the mean plasma sodium concentration was higher in non-survivors. In our study sample, there were very few occurrences of hypernatremia in both survivors and non-survivors. However, this was statistically different between the survivors and non-survivors. Since the incidence of hypernatremia was very low even in non-survivors, hypernatremia as clinical marker may not be valuable because the majority of sodium values were either normal or low in non-survivors. Higher sodium variability as compared to hypernatremia may be better associated with mortality from a clinical standpoint because sodium variability may more often reflect the degree of perturbation of sodium physiology than the infrequent occurrence of hypernatremia. Additionally, sodium variability may likely be present with or without hypernatremia, as such may be a more timely reflection in sodium metabolism and physiologic condition. Our multivariate analysis indicates that plasma sodium variability and not hypernatremia is an independent risk factor for mortality. This is probably due to the higher rate of dysnatremia in our study population and reflects the severe physiologic alterations that can occur following a severe burn injury. Additionally, early sodium variability may be more likely to be associated with death than late variability.

There are several limitations to this study. First, this was a retrospective cohort designed study. The effect of other variables specifically age and burn injury have a significant effect on mortality at admission, and it is likely the case that plasma sodium reflects changes in metabolism and physiology that develop over time and are a result of increased age or larger burn injury. Second, we were not able to assess the timing of acute renal failure with sodium variability. Acute renal failure was a diagnosis made by the treating physicians, and thus, variability likely exists in the timing and criteria used to make the diagnosis. A prospective study that analyzes the temporal relationship between sodium physiology and acute renal failure would provide the best analysis to determine the relationship between sodium and renal function. Third, we were not able to analyze the influence of intravenous fluid administration on sodium variability. All patients were resuscitated based on the Parkland formula (4 ml per kg per %TBSA); however, adjustments of fluid rates for resuscitation may have been made due to a number of factors. Delays in treatment, severity or cause of burn injury, concomitant injuries, inhalation injury, chemical, or illicit substance ingestions can all alter the acute resuscitation of burn patients. Additionally, daily weights could not be included in our analysis because they were variably collected in the patient charts. All maintenance fluid rates were based on the patients’ weight and evaporative loss due to the burn injury; however, the treating clinical team adjusted these rates based on their clinical decisions and thus cannot be normalized in a way that is comparable. A prospective study would add greatly to the influence of intravenous fluid amounts and sodium variability. Fourth, our current study does not directly aid clinicians about dysnatremia or sodium variability. Sodium variability would be difficult for a clinician to determine in a patient given the need to analyze and calculate the variability over a given time period (e.g., 7 days). It is likely that two things are needed in order for sodium variability to become a clinically useful marker. One, a prospective study needs to be completed that established the relationship between sodium variability over different time periods with current and future physiologic conditions. Two, once established, sodium variability limits can then be presented with laboratory analysis or through clinical calculators that will provide clinicians with established parameters to aid in their assessments of a patient’s clinical condition. Despite these limitations, our analysis indicates that hypernatremia is more prevalent in burn patients who die and that increased plasma sodium variability may be a risk factor for death.

## Conclusions

Dysnatremias may be a marker for poor outcomes and increased mortality for severely burned patients. The etiology for dysnatremias is multifactorial for burn patients, and increased variability of plasma sodium concentrations may be associated with an increased risk of death.
